# 746 Nurse Driven Fluid Resuscitation in the Burn Center

**DOI:** 10.1093/jbcr/irac012.299

**Published:** 2022-03-23

**Authors:** Emily H Werthman, Carrie A Cox, Julie Caffrey

**Affiliations:** Johns Hopkins Bayview Medical Center, Lutherville, Maryland; Johns Hopkins Bayview Medical Center, Baltimore, Maryland; Johns Hopkins, Baltimore, Maryland

## Abstract

**Introduction:**

The prior practice of the burn center was to resuscitate burn injuries over 20% total body surface area (TBSA) using a provider led modified Brooke fluid resuscitation formula. In that model of fluid resuscitation, the burn center provider ordered an initial fluid rate and adjusted hourly, where appropriate based on nurse recorded outputs. In Q4 2020 a nurse-driven fluid resuscitation was implemented in the adult Burn Intensive Care Unit (BICU). The primary purpose of the survey research is to evaluate the effect of the nurse-driven fluid resuscitation on nurse and physician communication.

**Methods:**

Survey research was initiated in Q3 2020 with a pre-practice change survey for BICU staff. The 3-part survey included 10 questions. The post survey will be repeated in August and remain open through October or until 70% of participants have completed the survey, whichever comes first.

**Results:**

Paired t-tests will be used compare survey research results pre and post-protocol implementation. In the pre-implementation survey there was a response rate of 44% (11/25). The average years of experience in the burn center was 11.64 years (median 7, SD 10.66). All survey questions were asked based on a 5-point Likert scale with anchors of 1 “strongly disagree” and 5 “strongly agree.” Question 1 the average score was 3.67. Question 2 the average score was 3.5. Question 3 the average score was 3.58. Question 4 the average score was 3.33. Question 5 the average score was 3.5. Content analysis was used to explore responses to open-ended questions. Three themes were identified: training, lack of communication, and over-resuscitation.

**Conclusions:**

The pre-implementation survey revealed highest scores on nurses and physicians having a good map of each other’s skills and lowest on providers and nurses discussing ways to prevent errors. Content analysis also revealed common concerns about miscommunication and lack of resuscitation training leading to over resuscitation. Upon completion of the post-implementation survey, we anticipate reporting changes in low scoring questions. We look forward to reporting these results as part of this abstract.

